# Concomitant inhaled corticosteroid use and the risk of pneumonia in COPD: a matched-subgroup *post hoc* analysis of the UPLIFT® trial

**DOI:** 10.1186/s12931-018-0874-0

**Published:** 2018-10-05

**Authors:** Donald P. Tashkin, Marc Miravitlles, Bartolomé R. Celli, Norbert Metzdorf, Achim Mueller, David M. G. Halpin, Antonio Anzueto

**Affiliations:** 1 Department of Medicine, David Geffen School of Medicine, University of California Los Angeles, 10833 Le Conte Avenue, Los Angeles, CA 90095-1690 USA; 2 Pneumology Department, Hospital Universitari Vall d’Hebron, CIBER de Enfermedades Respiratorias (CIBERES), Barcelona, Spain; 3 Brigham and Women’s Hospital, Boston, MA USA; 4 TA Respiratory/Biosimilars, Boehringer Ingelheim International GmbH, Ingelheim am Rhein, Germany; 5 Biostatistics and Data Sciences Europe, Boehringer Ingelheim Pharma GmbH & Co. KG, Biberach an der Riss, Germany; 6 Royal Devon and Exeter Hospital, Exeter, UK; 7 Pulmonary/Critical Care, University of Texas, and South Texas Veterans Health Care System, San Antonio, TX USA

**Keywords:** COPD, Pneumonia, Inhaled corticosteroids, Fluticasone propionate, UPLIFT®

## Abstract

**Background:**

Use of inhaled corticosteroids (ICS) increases the risk of pneumonia in chronic obstructive pulmonary disease (COPD), but the magnitude of risk with different ICS remains unclear.

**Methods:**

A *post hoc* analysis of the 4-year UPLIFT® trial to assess whether pneumonia risk differed by type of ICS (fluticasone propionate [FP], other ICS, or no ICS) in permanent users (defined by use until end of study) or in users at baseline (sensitivity analysis).

**Results:**

For the permanent-users analysis, 825 patients receiving FP throughout the trial, 825 patients receiving other ICS and 825 patients not receiving ICS were matched on relevant baseline features 1:1:1. A significantly greater risk of pneumonia was observed for FP versus no ICS: the hazard ratio (HR) for risk of pneumonia was 1.33 (95% confidence interval [CI] 1.00, 1.75; *p* = 0.046) and the rate ratio (RR) was 1.58 (95% CI 1.05, 2.37; *p* = 0.028). A greater risk was also found for FP versus other ICS: HR 1.28 (95% CI 0.97, 1.68; *p* = 0.078) and RR 1.48 (95% CI 1.00, 2.19; *p* = 0.049). A higher proportion of patients on FP were hospitalized with pneumonia (7.9%) versus other ICS (6.7%) or no ICS (5.9%). Whilst other ICS use was associated with the highest number of fatal pneumonia events, the total number of fatal pneumonia incidents was low. A similar pattern was observed in the sensitivity analyses, which included 4002 patients on different treatments at baseline (FP, other ICS, and no ICS) and considered potential switches during the study.

**Conclusion:**

The results support existing evidence of an increased pneumonia risk with FP use compared with other ICS and no ICS use in patients with COPD. Healthcare professionals should evaluate the risk–benefit ratio of using ICS when making treatment decisions with their patients.

**Trial registration:**

*Post hoc* analysis of UPLIFT®. ClinicalTrials.gov number: NCT00144339. Retrospectively registered September 2, 2005.

**Electronic supplementary material:**

The online version of this article (10.1186/s12931-018-0874-0) contains supplementary material, which is available to authorized users.

## Plain english summary

People with chronic obstructive pulmonary disease (COPD) who take inhaled corticosteroids (ICS) have a higher risk of pneumonia than those who don’t. However, it is not clear how big this risk is.

To help answer this question, we looked at findings from UPLIFT®, a large study of patients with COPD. We assessed whether ICS use affected pneumonia risk, and whether different types of ICS (fluticasone propionate or other types of ICS) had different effects. Unlike past investigations, we only included people who remained on the same type of ICS for the whole study, and compared these carefully to people who didn’t take any ICS but were otherwise similar. Therefore, any change in the risk of pneumonia was likely due to the ICS treatment rather than other potential effects, such as differences in disease severity between patients.

The results suggest that people taking fluticasone propionate have roughly 33% greater risk of pneumonia compared with those not taking ICS. They also have a 28% greater risk compared with people taking other ICS.

Overall, this study agrees with the results of other studies, and suggests that fluticasone propionate increases the risk of pneumonia compared with other ICS or no ICS in people with COPD. This is important because many people with COPD use fluticasone propionate. This study can help doctors to decide on the most appropriate treatment for people with COPD who are at risk of pneumonia.

## Background

Inhaled corticosteroids (ICS) are widely used for the treatment of chronic obstructive pulmonary disease (COPD) [1]. The Global Initiative for Chronic Obstructive Lung Disease (GOLD) report currently positions ICS in combination with bronchodilators as a second-line option to reduce exacerbation rates in patients in GOLD groups C and D who have experienced frequent exacerbations [2]. Despite this, it has been estimated that more than 70% of patients with COPD are treated with high doses of ICS, including those at low risk of exacerbations [3].

Recent randomized controlled trials suggest that ICS can be discontinued in some patients with stable COPD for whom ICS treatment may not be indicated without compromising safety and efficacy [4]. Moreover, the therapeutic benefits of widespread use of ICS remain controversial [3, 5–11]. High doses potentially result in systemic effects that could lead to glaucoma, cataracts, adrenal suppression, osteoporosis, bone fractures, skin bruising, and diabetes mellitus [4, 7, 8, 12–16].

While evidence for some of these adverse effects is weak, there is strong support (mainly from large clinical trials, population-based studies, and systematic reviews) for a significant increase in the risk of pneumonia in patients with COPD prescribed ICS (alone or in combination) [5, 6, 12, 17–24]. Published data in trials of up to 3 years in duration have shown an increased risk of pneumonia associated with ICS therapy in patients with COPD [19, 24, 25]. In light of this increased risk, it has been suggested that the use of ICS therapy should be restricted to the minority of patients with COPD who are likely to be particularly responsive to ICS therapy and in whom the benefits of treatment outweigh the risks [4]. Several studies suggest that the magnitude of pneumonia risk varies between types of ICS, with the greatest risk associated with fluticasone propionate (FP) [5, 6, 18, 22, 26]. However, there is conflicting evidence regarding the risk of pneumonia associated with different types of ICS, and this adds complexity when making treatment decisions [22, 27].

Interpretation of the clinical significance of many of the previous analyses of pneumonia risk in COPD is limited by the fact that they are from short-term studies [12] or that they only record the type of ICS use at baseline [28], and thus do not provide information on patients who remain on ICS in the long term. However, these issues can be overcome in subgroup analyses of the 4-year Understanding Potential Long-term Impacts on Function with Tiotropium (UPLIFT®) study, which permitted inhaled ICS use during both the run-in and treatment periods [29].

The UPLIFT® study included a large population of patients with COPD over 4 years [29]. Reflecting a close-to-real-life scenario, the patients were permitted to continue using respiratory medications, with the exception of other anticholinergics. This provides a large data set, in which we can investigate the effects of different types of ICS and their prescribing patterns, and how this relates to pneumonia outcomes. The initial study results showed similar overall risk of pneumonia during treatment with tiotropium 18 μg and placebo (risk 14.5% and 13.9%, respectively; relative risk [corrected for exposure] 0.96 [95% confidence interval (CI) 0.84, 1.10]) [29]. However, the original analysis of the study did not evaluate the risk of pneumonia in the subgroup of patients receiving concomitant ICS versus those without ICS throughout the duration of the trial, nor did it look at the individual effects of FP. A previous retrospective subgroup analysis of the UPLIFT® study data found that incidence rates of pneumonia were significantly higher in patients taking ICS, and that this increase was mainly associated with patients using FP (versus other ICS) at randomization [28]. However, this previous analysis had an uneven distribution of baseline characteristics between subgroups, which made interpretation of the results difficult.

We conducted this current *post hoc* analysis of the UPLIFT® study on pooled treatment groups to assess whether the risk of pneumonia in UPLIFT® differed according to the type of ICS used (FP, other ICS, and no ICS). We ran an analysis on matched subgroups of patients whom we know remained on the same ICS treatment throughout the study (permanent users), and repeated this type of analysis on the subgroups defined by baseline ICS use alone (two sensitivity analyses: FP, other ICS, and no ICS; and any ICS versus no ICS).

## Methods

### Study design and population

UPLIFT® was a 4-year, randomized, double-blind, placebo-controlled, parallel-group trial involving 5993 patients with moderate-to-very severe COPD, randomized to either tiotropium (*n* = 2987) or placebo (*n* = 3006) [29]. The trial evaluated the impact of tiotropium HandiHaler® 18 μg versus placebo on lung function, quality of life, exacerbations, and mortality.

Patients had a confirmed diagnosis of COPD and were aged ≥40 years, with a smoking history of ≥10 pack-years and a post-bronchodilator forced expiratory volume in 1 s (FEV_1_) ≤70% of the predicted value and an FEV_1_ ≤ 70% of forced vital capacity. Patients were excluded if they had a recent severe cardiac event or unstable COPD (exacerbation within 4 weeks), moderate or severe renal impairment, or other significant lung diseases. Pneumonia history was not assessed as an eligibility criterion for the study. Patients were permitted to use their usual background treatment for COPD (including ICS), except for other inhaled anticholinergics. Medication could be adjusted by the treating physician during the 4-year study duration; however, dose was not captured at baseline or during the study. For this analysis, the route of administration was derived from brand names and indication for use.

Full details of the UPLIFT® methodology have been published previously [29, 30].

### *Post hoc* analysis of pneumonia events

This post hoc analysis included ICS-treated patients matched with patients who had not received ICS during the UPLIFT® trial. Pneumonia risk was derived from general adverse event reports while patients were receiving a study drug (up to and including the last day of a study drug). Treatment arms were pooled for this analysis.

A diagnosis of pneumonia was determined by investigator-reported adverse events based on evidence of typical symptoms and clinical findings, together with chest radiology (where available) and laboratory findings. A mortality adjudication committee, comprised of members external to the study sponsor and of those involved in the conduct of the trial, adjudicated all reported deaths in the UPLIFT® trial; this committee provided a consistent, systematic, and independent assessment of the primary cause of death (e.g. fatal pneumonia).

### Statistical analysis

The study treatments – tiotropium and placebo – were pooled for the analyses, as there was no difference in risk of pneumonia between these treatment groups in the original analyses [31].

For the initial analysis, patients were classified by type of ICS use during the course of the study. Patients using FP throughout the study period were classified as “permanent FP users”. Patients using ICS from baseline to end of treatment (but not FP) were classified as “permanent users of other ICS” (mostly budesonide or beclomethasone). Patients in this “permanent users of other ICS” group could switch between different types of ICS, but not to FP or no ICS; 684 out of the 825 patients (82.9%) in this group did not switch to nor added another ICS. Those patients not taking any ICS during the course of the study were classified as “permanent no ICS users”.

A secondary sensitivity analysis was carried out on subgroups defined by type of ICS use at baseline (FP, other ICS, or no ICS); a third analysis investigated ICS use (all types) versus no ICS use at baseline. Furthermore, the number of pneumonia events was also analyzed according to the type of ICS use (FP, other ICS, or no ICS) reported at the last clinic visit before the first pneumonia event. All the patients in these latter analyses did not necessarily receive this background treatment regimen for the entire duration of the trial.

In order to minimize potential bias caused by differences in baseline characteristics between subgroups, patients were matched in a 1:1:1 ratio using the following baseline features: race, age (± 5 years), FEV_1_% predicted (± 5% predicted), GOLD stage, emphysema diagnosis, and antibiotic usage during the year prior to study start. The triplets of patients were generated randomly until no further matched triplets could be identified. Further matching processes were conducted for use of FP, other ICS or no ICS use at study baseline, and for ICS use versus no ICS use (secondary analyses).

For all analyses, Cox regression with matching factors as covariates was used to calculate hazard ratios (HR) and 95% CIs comparing time to first pneumonia, pneumonia requiring hospitalization, and pneumonia resulting in death between the matched subgroups. Annual rates, rate ratios (RR), and respective 95% CIs were calculated for pneumonia events and hospitalizations due to pneumonia using a Poisson regression model with matching factors as covariates.

## Results

### Analysis populations

Of the 5993 trial participants in the UPLIFT® study, a total of 3700 (61.7%) patients were receiving ICS at baseline. The first analyses of the matched subgroups of permanent users comprised 2475 patients (825 permanent FP users, 825 permanent users of other ICS, and 825 no ICS users). In the second analyses, which evaluated patients by type of ICS use at baseline, 4002 patients were matched, with 1334 patients per subgroup (FP, other ICS, and no ICS). Finally, 3948 patients were included in the analysis of any ICS versus no ICS use at baseline, with 1974 in each matched subgroup.

The demographic and baseline characteristics of the first two sets of matched subgroups – permanent users and users at baseline – are shown in Table 1. Corresponding data for the third analyses (patients receiving ICS versus no ICS at baseline) are provided in the data supplement (Additional file 1: Table S1). Baseline characteristics of the subgroups, including weight, height, and body mass index (BMI), were generally similar. The average age was approximately 65 years among the patients, and three-quarters of participants were male. Patients were mostly overweight (BMI > 25 kg/m^2^) and approximately one-third were current smokers. There was a higher proportion of current smokers in the permanent and baseline no-ICS groups than in the other groups. Over half of the patients had severe (GOLD Stage III) or very severe (GOLD Stage IV) COPD. There was also a lower proportion of patients receiving anticholinergics at baseline in the permanent and baseline no-ICS groups than in the other groups.

**Table 1:** Baseline characteristics of patients in matched subgroups by type of ICS use

	Permanent users			Use at baseline		
	FP (*n* = 825)	Other ICS (*n* = 825)	No ICS (*n* = 825)	FP (*n* = 1334)	Other ICS (*n* = 1334)	No ICS (*n* = 1334)
Male, *n* (%)	570 (69.1)	620 (75.2)	624 (75.6)	942 (70.6)	1014 (76.0)	996 (74.7)
Age, years, mean (SD)	65.0 (8.0)	64.9 (8.0)	64.8 (7.8)	64.9 (8.0)	64.9 (7.9)	64.8 (7.8)
Height, cm, mean (SD)	169.81 (9.2)	169.71 (8.6)	170.30 (8.5)	169.52 (9.0)	169.79 (8.4)	169.85 (8.7)
Weight, kg, mean (SD)	77.82 (16.7)	75.09 (15.9)	76.08 (18.1)	76.87 (16.7)	75.41 (16.3)	75.78 (17.9)
Body mass index, kg/m^2^, mean (SD)	26.89 (5.0)	25.97 (4.6)	26.10 (5.3)	26.65 (5.0)	26.05 (4.8)	26.15 (5.4)
Race, *n* (%)						
White	808 (97.9)	808 (97.9)	808 (97.9)	1291 (96.8)	1291 (96.8)	1291 (96.8)
Black	–	–	–	1 (0.1)	1 (0.1)	1 (0.1)
Asian	17 (2.1)	17 (2.1)	17 (2.1)	42 (3.1)	42 (3.1)	42 (3.1)
Current smoker, *n* (%)	191 (23.2)	216 (26.2)	298 (36.1)	316 (23.7)	347 (26.0)	473 (35.5)
Non-inhaled steroid use, *n* (%)	83 (10.1)	78 (9.5)	52 (6.3)	129 (9.7)	114 (8.5)	74 (5.5)
Anticholinergic use, *n* (%)	422 (51.2)	372 (45.1)	308 (37.3)	692 (51.9)	637 (47.8)	542 (40.6)
GOLD stage, *n* (%)						
II	395 (47.9)	395 (47.9)	395 (47.9)	621 (46.6)	621 (46.6)	621 (46.6)
III	371 (45.0)	371 (45.0)	371 (45.0)	608 (45.6)	608 (45.6)	608 (45.6)
IV	59 (7.2)	59 (7.2)	59 (7.2)	105 (7.9)	105 (7.9)	105 (7.9)
FEV_1_, L, mean (SD)	1.11 (0.4)	1.12 (0.4)	1.13 (0.4)	1.09 (0.4)	1.10 (0.4)	1.10 (0.4)
FEV_1_, % predicted, mean (SD)	39.72 (11.4)	39.80 (11.5)	39.78 (11.4)	39.14 (11.5)	39.10 (11.4)	39.17 (11.4)
FVC, L, mean (SD)	2.67 (0.9)	2.70 (0.8)	2.65 (0.8)	2.63 (0.9)	2.67 (0.8)	2.62 (0.8)
FVC, % predicted, mean (SD)	76.20 (18.6)	75.88 (17.4)	73.65 (17.0)	74.99 (18.2)	74.91 (17.5)	73.68 (17.4)
FEV_1_/FVC, mean (SD)	0.42 (0.1)	0.42 (0.1)	0.43 (0.1)	0.42 (0.1)	0.42 (0.1)	0.43 (0.1)
Post-bronchodilator FEV_1_, L, mean (SD)	1.34 (0.4)	1.35 (0.4)	1.37 (0.4)	1.32 (0.4)	1.33 (0.4)	1.34 (0.4)
Post-bronchodilator FEV_1_, % predicted, mean (SD)	48.06 (12.1)	47.90 (12.1)	48.09 (12.0)	47.62 (12.3)	47.30 (12.3)	47.68 (12.4)

*Abbreviations: FEV*_*1*_ forced expiratory volume in 1 s, *FP* fluticasone propionate, *FVC* forced vital capacity, *GOLD* Global Initiative for Chronic Obstructive Lung Disease, *ICS* inhaled corticosteroid, *SD* standard deviation. Patients within each group were matched by race, age (± 5 years), FEV_1_% predicted (± 5% predicted), GOLD stage, emphysema diagnosis, and courses of antibiotics during the previous year

### Risk of pneumonia

#### Permanent-user analysis

**Fig. 1:**
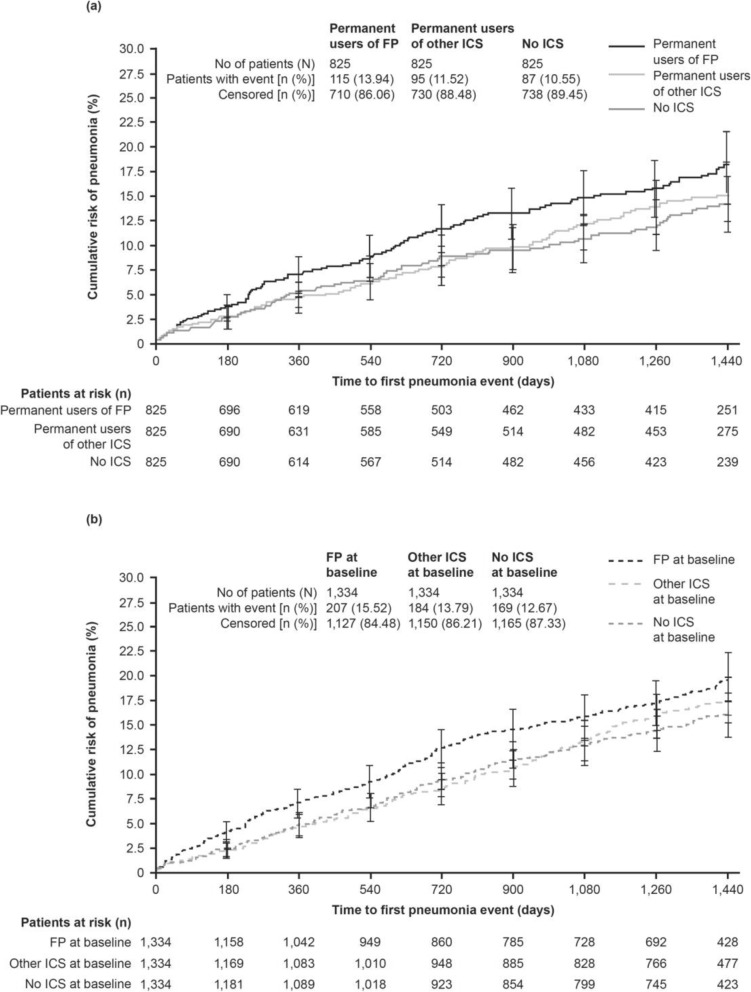
Time to first pneumonia in patients receiving ICS (FP, other, no): **a**) Permanent (**b**) Baseline. *Abbreviations:* FP: fluticasone propionate; ICS: inhaled corticosteroid

The results of the first analysis show that the time to first pneumonia was shorter and the rate of pneumonia (per patient-year) was greater in permanent users of FP compared with no ICS (HR 1.33 [95% CI 1.00, 1.75; *p* = 0.046] and RR 1.58 [95% CI 1.05, 2.37; *p* = 0.028], respectively) and compared with other ICS (HR 1.28 [95% CI 0.97, 1.68; *p* = 0.078] and RR 1.48 [95% CI 1.00, 2.19; *p* = 0.049], respectively; Table 2). However, HRs and RRs of pneumonia events did not differ between permanent users who received other ICS and patients who received no ICS (Table 2; Fig. 1a).

**Table 2:** Risk of pneumonia by type of ICS in permanent users

	Treatment throughout study			Comparison		
	FP	Other ICS	No ICS	FP versus other ICS HR* or RR^†^ (95% CI); *p-*value	FP versus no ICS HR* or RR^†^ (95% CI); *p*-value	Other ICS versus no ICS HR* or RR^†^ (95% CI); *p-*value
Permanent users, *n*	825	825	825	–	–	–
Patients with pneumonia events, *n* (%)^#^	115 (13.9)	95 (11.5)	87 (10.5)	HR 1.28 (0.97, 1.68); *p* = 0.078	HR 1.33 (1.00, 1.75); *p* = 0.046	HR 1.03 (0.77, 1.38); *p* = 0.830
Pneumonia events, *n*	159	111	98	–	–	–
Adjusted rate of pneumonia events (per patient-year), mean (95% CI)	0.10 (0.07, 0.14)	0.07 (0.04, 0.10)	0.06 (0.04, 0.10)	RR 1.48 (1.00, 2.19); *p* = 0.049	RR 1.58 (1.05, 2.37); *p* = 0.028	RR 1.07 (0.69, 1.65); *p* = 0.773
Patients with hospitalized pneumonia events, *n* (%)	65 (7.9)	55 (6.7)	49 (5.9)	HR 1.24 (0.86, 1.77); *p* = 0.249	HR 1.30 (0.90, 1.88); *p* = 0.168	HR 1.05 (0.71, 1.54); *p* = 0.802
Hospitalized pneumonia events, *n*	78	64	51	–	–	–
Adjusted rate of hospitalized pneumonia events (per patient-year), mean (95% CI)	0.05 (0.03, 0.08)	0.04 (0.03, 0.07)	0.04 (0.02, 0.06)	RR 1.25 (0.78, 2.02); *p* = 0.355	RR 1.47 (0.88, 2.44); *p* = 0.141	RR 1.17 (0.69, 1.99); *p* = 0.560
Patients with pneumonia resulting in death, *n* (%)	7 (0.85)	13 (1.58)	9 (1.09)	HR 0.54 (0.22, 1.36); *p* = 0.194	HR 0.75 (0.28, 2.01); *p* = 0.567	HR 1.38 (0.59, 3.23); *p* = 0.460

*Abbreviations: CI* confidence interval, *FEV*_*1*_ forced expiratory volume in 1 s, *FP* fluticasone propionate, *GOLD* Global Initiative for Chronic Obstructive Lung Disease, *HR* hazard ratio, *ICS* inhaled corticosteroid, *RR* rate ratio. Matched-subgroup population. *Cox regression analysis with ICS use on-treatment and matching factors as covariates. ^†^Poisson regression with ICS use on-treatment and matching factors (age, FEV_1_% predicted, GOLD stage, emphysema diagnosis, and courses of antibiotics) as covariates. ^#^In this analysis, 23 patients treated permanently with FP received the treatment for non-pulmonary (mostly nasal) indication only; three of these patients had a pneumonia event during the study. In the subgroup permanently treated with other ICS, these numbers were 10 and 3, respectively

**Fig. 2:**
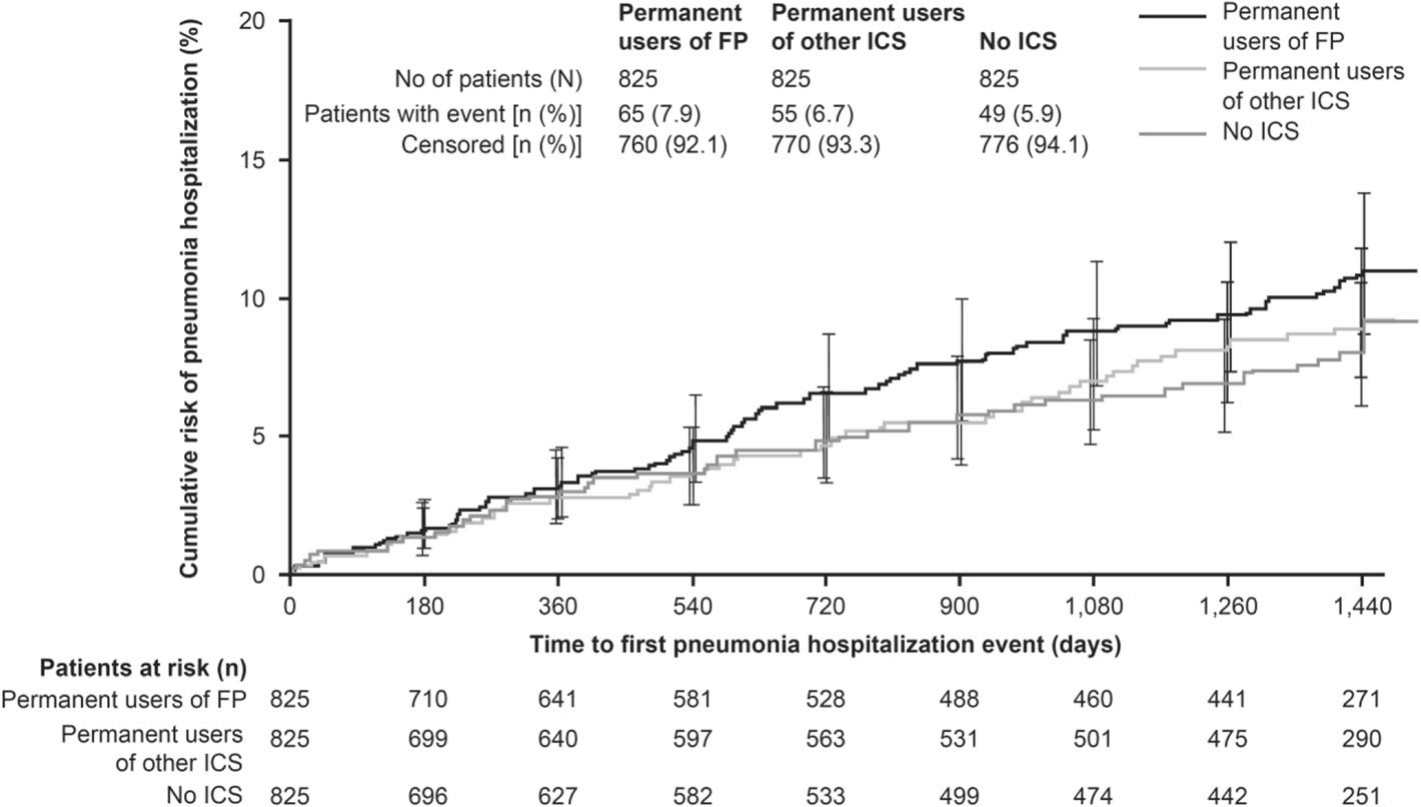
Time to first pneumonia hospitalization by type of ICS (permanent users). *Abbreviations:* FP: fluticasone propionate; ICS: inhaled corticosteroid

The risk of hospitalized pneumonia, based on time to first pneumonia hospitalization, was also greater in permanent users of FP treatment compared with other ICS and no ICS (Fig. 2). A numerically higher proportion of permanent FP users was hospitalized with pneumonia events (7.9%) compared with patients treated with no ICS (5.9%) or other ICS (6.7%; Table 2). Based on small patient numbers, a numerically higher proportion of patients receiving other ICS (*n* = 13, 1.58%) had fatal pneumonia events than those receiving no ICS (*n* = 9, 1.09%) or users of FP (*n* = 7, 0.85%) (Table 2).

#### Matched-subgroups analysis by type of ICS use at baseline

A similar pattern was observed in this second analysis, which evaluated patients according to their ICS use at the start of the study. Patients treated with FP at baseline had an increased risk of pneumonia, in terms of time to event, versus patients not on ICS (HR 1.30 [95% CI 1.06, 1.59; *p* = 0.012]; Table 3), with a 20% higher risk with FP than for other ICS (HR 1.20 [95% CI 0.99, 1.47; *p* = 0.067]; Table 3; Fig. 1b). Likewise, the annual rate of pneumonia was highest in patients receiving FP versus no ICS (RR 1.37 [95% CI 1.06, 1.78; *p* = 0.015]) or other ICS (RR 1.35 [95% CI 1.05, 1.74; *p* = 0.020]) at baseline (Table 3). Only minor differences were observed between pneumonia events in patients on other ICS and those on no ICS (Table 3; Fig. 1b).

**Table 3:** Risk of pneumonia by type of treatment (FP, other ICS, no ICS use) at baseline

	Treatment at baseline			Comparison		
	FP	Other ICS	No ICS	FP versus other ICS HR* or RR^†^ (95% CI); *p-*value	FP versus no ICS HR* or RR^†^ (95% CI); *p*-value	Other ICS versus no ICS HR* or RR^†^ (95% CI); *p-*value
Use at baseline, *n*	1334	1334	1334	–	–	–
Patients with pneumonia event, *n* (%)^#^	207 (15.5)	184 (13.8)	169 (12.7)	HR 1.20 (0.99, 1.47); *p* = 0.067	HR 1.30 (1.06, 1.59); *p* = 0.012	HR 1.08 (0.88, 1.33); *p* = 0.467
Pneumonia events, *n*	286	220	214	–	–	–
Adjusted rate of pneumonia events (per patient-year), mean (95% CI)	0.10 (0.08, 0.12)	0.07 (0.06, 0.09)	0.07 (0.05, 0.09)	RR 1.35 (1.05, 1.74); *p* = 0.020	RR 1.37 (1.06, 1.78); *p* = 0.015	RR 1.02 (0.77, 1.34); *p* = 0.907
Patients with hospitalized pneumonia events, *n* (%)	140 (10.5)	114 (8.5)	100 (7.5)	HR 1.31 (1.02, 1.68); *p* = 0.032	HR 1.48 (1.14, 1.91); *p* = 0.003	HR 1.13 (0.86, 1.47); *p* = 0.382
Hospitalized pneumonia events, *n*	172	133	121	–	–	–
Adjusted rate of hospitalized pneumonia events (per patient-year), mean (95% CI)	0.06 (0.04, 0.08)	0.04 (0.03, 0.06)	0.04 (0.03, 0.05)	RR 1.34 (0.99, 1.81); *p* = 0.055	RR 1.46 (1.07, 1.98); *p* = 0.016	RR 1.09 (0.79, 1.51); *p* = 0.607
Patients with pneumonia resulting in death, *n* (%)	8 (0.60)	18 (1.35)	15 (1.12)	HR 0.46 (0.20, 1.05); *p* = 0.066	HR 0.55 (0.23, 1.30); *p* = 0.174	HR 1.20 (0.61, 2.39); *p* = 0.595

*Abbreviations: CI* confidence interval, *FEV*_*1*_ forced expiratory volume in 1 s, *FP* fluticasone propionate, *GOLD* Global Initiative for Chronic Obstructive Lung Disease, *HR* hazard ratio, *ICS* inhaled corticosteroid, *RR* rate ratio. Matched-subgroup population. *Cox regression analysis with ICS use at baseline and matching factors as covariates. ^†^Poisson regression with ICS use at baseline and matching factors (age, FEV_1_% predicted, GOLD stage, emphysema diagnosis, and courses of antibiotics) as covariates. ^#^32 patients treated with FP at baseline received this treatment for non-pulmonary (mostly nasal) indication only; four of these patients had a pneumonia event during the study. In the “other ICS” group, these numbers were 43 and 11, respectively

A higher proportion of patients treated with FP at baseline was hospitalized with pneumonia events (10.5%) compared with patients receiving no ICS (7.5%) or on other ICS (8.5%) at baseline. The risk of hospitalization due to pneumonia was greater in patients treated with FP at baseline compared with no ICS (HR 1.48 [95% CI 1.14, 1.91; *p* = 0.003] and RR 1.46 [95% CI 1.07, 1.98; *p* = 0.016], respectively) or other ICS (HR 1.31 [95% CI 1.02, 1.68; *p* = 0.032] and RR 1.34 [95% CI 0.99,1.81; *p* = 0.055], respectively). The HR and RR of hospitalized pneumonia events were slightly increased for other ICS versus no ICS use at baseline (Table 3). However, based on small patient numbers, numerically more patients receiving other ICS at baseline (*n* = 18, 1.35%) had fatal pneumonia events than those receiving no ICS (*n* = 15, 1.12%) or FP (*n* = 8, 0.60%) at baseline (Table 3).

#### ICS therapy proximate to the first pneumonia event (last visit before event)

As the UPLIFT® study allowed changes in the concomitant therapy of the patients, pneumonia events were also analyzed according to the type of ICS use (FP, other ICS, or no ICS) reported at the last clinic visit before the first pneumonia event. Table 4 shows the percentage of patients with a first pneumonia or hospitalized/fatal pneumonia event who received FP, other ICS, or no ICS at baseline, and also describes the percentage who were on these treatments at the last visit before the event occurred. All patients switching treatments prior to the event, as well as those who had no pneumonia event and switched at any time during the study, were counted in each respective group, thereby increasing the denominator compared with the patients in the baseline analysis so that all percentages are lower. Taking switches into account did not result in important changes in the overall results. The relative pattern of the frequency of pneumonia and hospitalized pneumonia events according to treatment was maintained (Table 4).

**Table 4:** Percentage of patients experiencing pneumonia events, stratified by treatment at baseline and last visit proximate to event

	Treatment		
	FP	Other ICS	No ICS
Patients with first pneumonia event (%)			
Treatment at baseline	15.5	13.8	12.7
Treatment at last visit before event^a^	12.9	10.1	8.6
First hospitalized pneumonia event (%)			
Treatment at baseline	10.5	8.5	7.5
Treatment at last visit before event^a^	8.3	6.6	5.2
Pneumonia event resulting in death (%)			
Treatment at baseline	0.6	1.3	1.1
Treatment at last visit before event^a^	0.6	0.9	0.7

*Abbreviations: FP* fluticasone propionate, *ICS* inhaled corticosteroid. Patients were matched at baseline (matched subgroup by baseline ICS use population, *N* = 4002). ^a^Percentages are calculated as patients with pneumonia events / patients taking treatment at any time prior to the event or, in those without event, at any time during the study

#### Matched-pairs analysis by any ICS versus no ICS use at baseline

Analyses based on patients treated with all types of ICS versus no ICS at baseline are reported in the data supplement (Additional file 1: Table S2, Figure S1). In summary, the risk of pneumonia events was increased in patients with ICS use at baseline as compared with those without ICS (HR 1.20 [95% CI 1.01, 1.42; *p* = 0.037]; RR 1.18 [95% CI 0.95, 1.48; *p* = 0.134]). The proportion of patients with pneumonia leading to hospitalization was higher in the ICS subgroup (8.9%) compared with the no-ICS subgroup (7.4%). Whilst the annual rate of hospitalized pneumonia (RR 1.17 [95% CI 0.89, 1.54; *p* = 0.251]) supports the decreased time to hospitalized pneumonia events with ICS use versus no ICS at baseline (HR 1.22 [95% CI 0.98, 1.52; *p* = 0.076]), the number of deaths from pneumonia did not differ by ICS use at baseline (HR 1.01 [95% CI 0.52, 1.98; *p* = 0.979]).

## Discussion

Despite recommendations for the use of ICS in combination with long-acting bronchodilators for the management of patients with frequent exacerbations [32], uncertainties about the efficacy and potential side effects of ICS therapies have led to concerns regarding their use [4]. In this *post hoc* analysis of the 4-year UPLIFT® trial, we assessed whether ICS use, and particularly FP, when taken long-term in the treatment of COPD was associated with a higher risk of pneumonia. Our findings indicate that long-term use of FP was associated with a 48% increased rate of pneumonia compared with other types of ICS, and a 58% increased risk compared with no ICS treatment. In this analysis, there was no significant difference between patients who received other ICS compared with patients who received no ICS, suggesting that FP may have a stronger association with pneumonia than alternative ICS therapies. These findings were also supported by two sensitivity analyses that investigated the risk of a pneumonia event by ICS treatment at baseline, and taking switches during the study into account.

In this analysis, the risk of pneumonia (based on time to first event and annual rate) was higher in patients treated with FP compared with other ICS or no ICS, and this was consistent whether patients were receiving those treatments permanently throughout the study or at baseline. Our results add to those of a previous retrospective *post hoc* analysis of UPLIFT®, which concluded that FP usage was associated with excess morbidity (including risk of pneumonia and increase in COPD exacerbations) compared with other ICS or no ICS [28]. In the previous analysis, the subgroups were not matched according to their baseline characteristics and permanent users were not analyzed; therefore, our analysis makes it unlikely that differences between the ICS groups may be due to different disease severity at baseline.

An increased risk of pneumonia with FP versus other ICS or no ICS has been reported previously by other randomized studies of FP-containing regimens in COPD [5, 6, 11, 18, 20, 28]. A review of 43 randomized controlled studies of COPD treatments found that fluticasone (either alone or in combination with long-acting β_2_-agonist [LABA] therapy) increased the risk of non-fatal serious adverse pneumonia events (requiring hospital admission) compared with placebo or LABA monotherapy [6]. Furthermore, fluticasone was associated with a higher risk of any pneumonia event (including community-treated cases) compared with the ICS budesonide, with an odds ratio of 1.86 (95% CI 1.04, 3.34) [6]. A relatively high risk of pneumonia with FP therapy compared with budesonide use was also reported by a large cohort study of more than 160,000 patients with COPD over 5 years of follow-up, specifically with regard to serious events [5]. The 2-year Investigating New Standards for Prophylaxis in Reduction of Exacerbations (INSPIRE) and the 3-year Towards a Revolution in COPD Health (TORCH) trials both studied high daily doses of FP (1000 μg per day) in COPD patients [18, 20]. In INSPIRE, the HR for time to first pneumonia was 1.94 (95% CI 1.19, 3.17) for the salmeterol/FP combination (SFC) versus tiotropium [20]; in TORCH, the HRs were 1.64 (95% CI 1.33, 2.02) for SFC versus placebo, and 1.53 (95% CI 1.24, 1.89) for FP versus placebo [18]. Additionally, in a meta-analysis of 54 randomized controlled studies including 61,551 patients with COPD, the two treatments that were shown to increase the risk of pneumonia compared with placebo were FP and SFC [22].

More recent studies have also looked at the association of fluticasone furoate (FF) with pneumonia; this is a different salt form compared with FP, with distinct properties [33]. Data for the association of pneumonia with FF from recent large-scale studies (the Salford Lung Study, *N* = 2799) [34] and the Study to Understand Mortality and MorbidITy in COPD [SUMMIT], *N* = 16,485) [35] are difficult to interpret. The Salford Lung Study did report a trend toward a higher mean number of serious pneumonia adverse events with FF/vilanterol (FF/VI) in the subgroup of patients who had not been receiving ICS at baseline [34]. However, in patients receiving ICS at study baseline, the study was effectively comparing FF/VI versus other ICS (including FP), and showed no notable difference between the two groups. In SUMMIT, the results were also mixed, with no obvious increase in pneumonia rates for the comparison of the FF-containing arms versus placebo; however, compared with the VI arms, the rates per 100 patient-years were 3.9 for the FF/VI combination and 4.2 for FF monotherapy, compared with 2.8 for VI monotherapy [35]. A greater risk of pneumonia with FF/VI compared with VI monotherapy was also reported previously in an analysis of two 1-year studies of patients with moderate-to-very severe airflow limitation and at least one COPD exacerbation in the previous year [21]. Most recently, in the FULFIL trial, a head-to-head comparison showed that pneumonia was reported in 20/911 (2.2%) patients in the FF arm compared with 7/899 (0.8%) in the budesonide arm (*p* < 0.01) [36, 37].

In a nested case-controlled study with a large cohort of 175,906 patients with COPD, current use of ICS (within the last 60 days) was associated with a significant 70% increase in the risk of being hospitalized with severe pneumonia [17]. This effect was even greater with higher doses of ICS (equivalent to 1000 μg fluticasone per day) [17]. Although the impact of particular ICS therapies was not assessed, this suggests that patients with COPD receiving ICS in general practice should be closely monitored for pneumonia events and treated promptly to avoid the need for secondary care.

In our *post hoc* analysis, a higher proportion of patients on FP were hospitalized with pneumonia events compared with patients treated with other ICS or no ICS, and the annual rate of hospitalization was also highest with FP. The sensitivity analysis by type of ICS use at baseline supported the higher annual rate of hospitalization with FP. This suggests that patients with COPD receiving FP may require particularly careful surveillance.

With regard to pneumonia events leading to death, the numbers with FP were somewhat smaller than with other ICS. However, the overall numbers of fatal pneumonia events in UPLIFT® are too small to adequately address this topic here.

It is unclear why patients receiving FP may be more likely to experience pneumonia (including events requiring hospitalization) relative to those receiving alternative ICS. It has been suggested that the difference between FP and budesonide is the longer retention of FP in the airways [38]. FP can persist for hours in the airway lining fluid, whereas budesonide is absorbed away within minutes. In this way, FP can suppress the immune system, enhance susceptibility to respiratory infections, and increase the load of pathogenic microbiome in the airways and lungs, leading to pneumonia in the weeks following an unresolved exacerbation [38]. Another potential explanation is due to the dosing of FP. ICS are often prescribed to patients with COPD at high doses, and the association between pneumonia and ICS use may be dose-related, with high doses of ICS having the greatest risk of pneumonia compared with low and medium doses [19, 39]. As FP is likely to be given at higher doses than other ICS, this could be one explanation for the results seen here, although other data have shown that an increased risk of pneumonia can be associated even with low doses of ICS [40], and therefore dose may not fully explain the association with pneumonia.

It has been reported that the risk of pneumonia is increased in patients with more advanced COPD or severe airflow limitation [41]. Therefore, for the purposes of our *post hoc* analysis, patients were matched by baseline FEV_1_% predicted and GOLD stage, thus avoiding confounding by severity of underlying disease. Just over half of the patients included in the present analyses had severe (GOLD Stage III) to very severe (GOLD Stage IV) COPD, similar to the primary study demographic [29]. An observational study of patients hospitalized for pneumonia reported that of 4121 community-acquired pneumonia episodes, 23.9% occurred in patients with COPD, and 58% of these patients were GOLD Stage III or IV [42]. Given the likelihood of an increased risk of pneumonia with both the progression of disease and ICS use (particularly FP, as supported by the current and previously published analyses of large-scale COPD trials), physicians need to consider pneumonia as part of the risk–benefit ratio of ICS use (and in the choice of ICS) relative to disease severity prior to prescribing [7].

This analysis of UPLIFT® has strengths and limitations. UPLIFT® provides a large data set over 4 years that enables evaluation of treatment effects on rare adverse events. Unlike other landmark trials of COPD, patients were permitted to continue using their background therapy (including ICS) and also have their treatment adapted, reflecting real-life clinical practice. Information on the type(s) of ICS was collected at baseline and throughout the study, allowing comparison of the effects of different types of ICS on pneumonia risk. Furthermore, the long study duration allowed time for potential ICS-specific side effects to manifest themselves. However, the UPLIFT® trial was not designed to study pneumonia as a specific event; therefore, it was not statistically powered to detect differences in pneumonia between the subgroups. In addition, since the dosage of FP or other ICS at baseline and at the end of treatment was not captured during the UPLIFT® study, and as dosage will vary between countries, it was not possible to evaluate the treatment dose-response. Not all of the pneumonia events during UPLIFT® were verified by chest X-ray, although patients with a hospitalization for suspected pneumonia were likely to have their diagnosis confirmed radiographically. Furthermore, due to the overlap in presentation of pneumonia and exacerbations in a primary care setting, there can be difficulties in distinguishing between these. Here, this may have led to biases, particularly as patients were not matched for previous exacerbation history in this analysis. Additionally, in this analysis, smoking status was not included in the matching baseline features, as doing so resulted in a considerably smaller sample size and there was a difference in smoking status between subgroups. There was also a greater proportion of anticholinergic users at baseline in the FP group than in the no-ICS group. A pooled safety analysis showed that tiotropium does not have a significant effect on pneumonia [31], but if tiotropium’s beneficial effect on exacerbations reduced pneumonia reporting in the FP group, this would have attenuated the apparent effect of FP on pneumonia rates. Finally, it is possible that FP treatment before the study was a marker of a patient’s sensitivity to pneumonia, and this could not be balanced by matching the patients. However, it is difficult to see why this would be different for other ICS, which showed a smaller risk of pneumonia than FP.

## Conclusions

This *post hoc* analysis of the UPLIFT® trial indicated that use of FP over 4 years was associated with an increased risk of pneumonia compared with no ICS treatment or other types of ICS. This did not appear to be confounded by factors associated with ICS treatment (such as disease severity), as a matched-subgroup analysis was conducted. In the current analysis, other ICS showed only numerical increases in pneumonia risk versus no ICS treatment.

The findings add to the existing evidence regarding pneumonia risk associated with ICS therapy and, in particular, long-term FP use in patients with COPD.

## Additional file

Additional file 1: Matched-pairs analysis by ICS versus no ICS use at baseline. (DOCX 453 kb)

## Abbreviations

BMI: Body mass index
CI: Confidence interval
COPD: Chronic obstructive pulmonary disease
FEV_1_: Forced expiratory volume in 1 s
FF: Fluticasone furoate
FP: Fluticasone propionate
GOLD: Global Initiative for Chronic Obstructive Lung Disease
HR: Hazard ratio
ICS: Inhaled corticosteroid
INSPIRE: Investigating New Standards for Prophylaxis in Reduction of Exacerbations
LABA: Long-acting β_2_-agonists
RR: Rate ratio
SFC: Salmeterol/fluticasone propionate combination
SUMMIT: Study to Understand Mortality and MorbidITy
TORCH: Towards a Revolution in COPD Health
UPLIFT®: Understanding Potential Long-term Impacts on Function with Tiotropium
VI: Vilanterol
