# Supplementary material for: Concomitant inhaled corticosteroid use and the risk of pneumonia in COPD: a matched-subgroup post hoc analysis of the UPLIFT® trial
Source: Respir Res. 2018 Oct 5;19:196. doi: 10.1186/s12931-018-0874-0 (PMC6173940; doi:10.1186/s12931-018-0874-0)
Supplement: Supplementary file 1 — Matched-pairs analysis by ICS versus no ICS use at baseline. (DOCX 453 kb) [file 12931_2018_874_MOESM1_ESM.docx]

**Data supplement**

**Matched-pairs analysis by ICS versus no ICS use at baseline.**

Analysis population.

**Table S1** Baseline characteristics of patients, according to matched subgroups by ICS use at baseline

|  | **Matched pairs by ICS use at baseline** | |
| --- | --- | --- |
|  | **ICS use**  **(*n*** **=** **1974)** | **No ICS use**  **(*n*** **=** **1974)** |
| Male, *n* (%) | 1384 (70.1) | 1489 (75.4) |
| Age, years, mean (SD) | 64.38 (8.3) | 64.26 (8.3) |
| Height, cm, mean (SD) | 169.56 (9.0) | 169.53 (8.6) |
| Weight, kg, mean (SD) | 76.74 (17.2) | 75.22 (17.9) |
| Body mass index, kg/m^2^, mean (SD) | 26.57 (5.1) | 26.04 (5.3) |
| Race, *n* (%) | | |
| White | 1849 (93.7) | 1849 (93.7) |
| Black | 18 (0.9) | 18 (0.9) |
| Asian | 107 (5.4) | 107 (5.4) |
| Current smoker, *n* (%) | 494 (25.0) | 732 (37.1) |
| Non-inhaled steroid use, *n* (%) | | |
| Yes | 173 (8.8) | 114 (5.8) |
| Anticholinergic use, *n* (%) | | |
| Yes | 965 (48.9) | 794 (40.2) |
| GOLD stage, *n* (%) | | |
| II | 1005 (50.9) | 1005 (50.9) |
| III | 836 (42.4) | 836 (42.4) |
| IV | 133 (6.7) | 133 (6.7) |
| FEV_1_, L, mean (SD) | 1.13 (0.4) | 1.15 (0.4) |
| FEV_1_, L, % predicted, mean (SD) | 40.54 (11.9) | 40.73 (11.8) |
| FVC, L, mean (SD) | 2.68 (0.9) | 2.66 (0.8) |
| FVC, % predicted, mean (SD) | 76.30 (18.0) | 74.97 (17.5) |
| FEV_1_/FVC ratio, mean (SD) | 0.43 (0.1) | 0.44 (0.1) |
| Post-bronchodilator FEV_1_, L, mean (SD) | 1.36 (0.4) | 1.38 (0.5) |
| Post-bronchodilator FEV_1_ % predicted, mean (SD) | 48.86 (12.3) | 49.05 (12.4) |

*Abbreviations:* FEV_1_: forced expiratory volume in 1 s; FVC: forced vital capacity; GOLD: Global Initiative for Chronic Obstructive Lung Disease; ICS: inhaled corticosteroid; SD: standard deviation. Patients within each group were matched by race, age (± 5 years), FEV_1_% predicted (± 5% predicted), GOLD stage, emphysema diagnosis, and courses of antibiotics during the previous year

**Table S2** Risk of pneumonia events in ICS versus no ICS subgroup at baseline

|  | **Matched pairs by ICS use at baseline** | | |
| --- | --- | --- | --- |
|  | **ICS use** | **No ICS use** | **HR* or RR^†^ (95% CI); *p*-value** |
| Patients, *n* | 1974 | 1974 |  |
| Patients with pneumonia event, *n* (%) | 291 (14.7) | 249 (12.6) | HR 1.20 (1.01, 1.42);  *p* = 0.037 |
| Pneumonia events, *n* | 365 | 309 | – |
| Adjusted rate of pneumonia events (per patient-year), mean (95% CI) | 0.09 (0.07, 0.11) | 0.07 (0.06, 0.10) | RR 1.18 (0.95, 1.48);  *p* = 0.134 |
| Patients with hospitalized pneumonia events, *n* (%) | 176 (8.9) | 147 (7.4) | HR 1.22 (0.98, 1.52);  *p* = 0.076 |
| Hospitalized pneumonia events, *n* | 203 | 174 | – |
| Adjusted rate of hospitalized pneumonia events (per patient-year), mean (95% CI) | 0.05 (0.04, 0.07) | 0.04 (0.03, 0.06) | RR 1.17 (0.89, 1.54);  *p* = 0.251 |
| Patients with pneumonia resulting in death, *n* (%) | 17 (0.9) | 17 (0.9) | HR 1.01 (0.52, 1.98);  *p* = 0.979 |

*Abbreviations:* CI: confidence interval; HR: hazard ratio; ICS: inhaled corticosteroid; RR: rate ratio. Matched-pairs population. *Cox regression analysis with ICS use at baseline and matching factors as covariates. ^†^Poisson regression with ICS use at baseline and matching factors as covariates

**Figure S1** Time to first pneumonia event in patients with ICS versus no ICS use at baseline


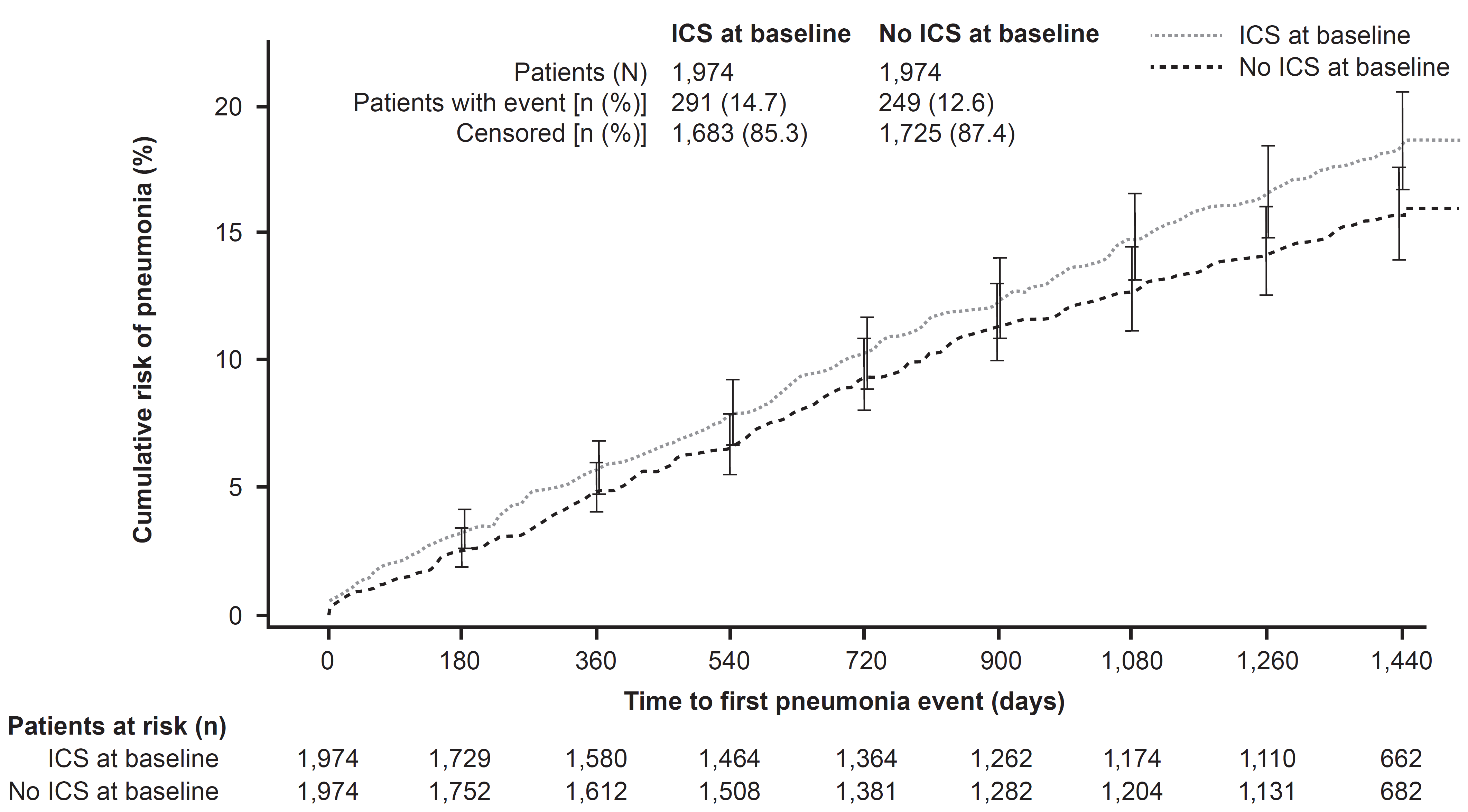


*Abbreviations:* ICS: inhaled corticosteroid
